# Supplementary material for: “Colorectal Cancer Care Quality in a Developing Country: Insights from a Comparison of Teaching and Non-teaching Hospitals in Iran”
Source: PLoS One. 2025 Sep 8;20(9):e0326796. doi: 10.1371/journal.pone.0326796 (PMC12416677; doi:10.1371/journal.pone.0326796)
Supplement: S1 Table — (DOCX) [file pone.0326796.s001.docx]

S1 Table: Comparing the censored and non-censored patients according to different patient-related variables among colorectal cancer patients in Iran.

| **Variables** | Group | Non-censored | Censored | P-value |
| --- | --- | --- | --- | --- |
| **Gender** | Male | 169 (41.2) | 241 (58.8) | 0.94 |
|  | Female | 107 (41.5) | 151 (58.5) |  |
| **age** | Mean (95%CI) | 58.9 (57.1-60.6) | 56.9 (55.6-58.1) | 0.06 |
| **Residential Status** | Urban | 223 (41.0) | 321 (59.0) | 0.11 |
|  | Suburban | 27 (54.0) | 23 (46.0) |  |
|  | Rural | 26 (35.1) | 48 (64.9) |  |
| **Topography** | Colon | 160 (43.2) | 210 (56.8) | 0.26 |
|  | Rectum | 116 (38.9) | 182 (61.1) |  |
| **Stage at diagnosis** | Stage I | 9 (50) | 9 (50) | 0.25 |
|  | Stage II | 15 (27.8) | 39 (72.2) |  |
|  | Stage III | 71 (40.3) | 105 (59.7) |  |
|  | Unknown | 21 (42.0) | 29 (58.0) |  |
| **Type of Hospital** | TCC | 158 (38.3) | 248 (61.7) | 0.06 |
|  | NTNC | 122 (45.9) | 144 (51.1) |  |
| **Adherence to the Guideline in Adjuvant chemotherapy** | Adherent | 161 (38.0) | 263 (62.0) | 0.15 |
|  | Not-adherent | 58 (46.0) | 68 (54.0) |  |
|  | Unknown | 28 (33.7) | 55 (66.3) |  |
