# Supplementary material for: “Colorectal Cancer Care Quality in a Developing Country: Insights from a Comparison of Teaching and Non-teaching Hospitals in Iran”
Source: PLoS One. 2025 Sep 8;20(9):e0326796. doi: 10.1371/journal.pone.0326796 (PMC12416677; doi:10.1371/journal.pone.0326796)
Supplement: S2 Table — (DOCX) [file pone.0326796.s002.docx]

S2 Table: Short-term mortality rate in TCCs vs NTNCs in colon and rectal cases in Iran

| **Mortality rate** | **Colon &  Rectum** | **TCCs**  N (%) | **NTNCs**  N (%) | **P-value** |
| --- | --- | --- | --- | --- |
| 30-day | Colon | 5 (3) | 10 (4.9) | 0.35 |
|  | Rectum | 8 (3.4) | 3 (4.8) | 0.61 |
| 60-day | Colon | 9 (5.4) | 12 (5.9) | 0.83 |
|  | Rectum | 9 (3.8) | 4 (6.4) | 0.38 |
| 90-day | Colon | 11 (6.6) | 15 (7.4) | 0.76 |
|  | Rectum | 9 (3.8) | 6 (9.5) | **0.07** |
| 180-day | Colon | 6 (3.6) | 12 (6) | 0.30 |
|  | Rectum | 7 (3) | 4 (6.4) | 0.21 |

TCC=Teaching Cancer Cener; NTNC=High-volume non-teaching non-cancer hospital
