# Supplementary material for: “Colorectal Cancer Care Quality in a Developing Country: Insights from a Comparison of Teaching and Non-teaching Hospitals in Iran”
Source: PLoS One. 2025 Sep 8;20(9):e0326796. doi: 10.1371/journal.pone.0326796 (PMC12416677; doi:10.1371/journal.pone.0326796)
Supplement: Attachment — Submitted filename: Rebuttel Letter_D-23-43777_kz_MRR.docx [file pone.0326796.s003.docx]

Kazem Zendehdel,

Professor at the Cancer Research Center,

Cancer Institute, Tehran University of Medical Sciences,

Tehran, Iran.

[kzendeh@tums.ac.ir](mailto:kzendeh@tums.ac.ir)

11-28-2024

Dear Dr. Sina Azadnajafabad,

We would like to express our gratitude to you and the reviewers for the insightful comments and suggestions on our manuscript titled "Bridging Gaps in Colorectal Cancer Care Quality in Developing Countries: Navigating Disparities with the Right Approach, Analytical Methods, and Minimum Essential Data (PONE-D-23-43777)."

We have carefully addressed each reviewer's comments and made the necessary revisions throughout the manuscript. In response to the constructive feedback from the four reviewers, we undertook extensive revisions in all sections. We provide highlighted and clean versions of the revised paper for your consideration. We hope the revised manuscript will be accepted for publication in PLOS ONE.

Regarding data availability, we have provided further justification for why the data cannot be shared publicly. Since the patients did not consent to the public distribution of their data, and the Ethics Committee of Tehran University of Medical Sciences (TUMS) has not granted such rights to researchers, the data from this study cannot be published in public databases due to privacy concerns. However, the data may be available for research purposes upon reasonable request from the corresponding author (K.Z.) or the Chair of the Cancer Research Institute at TUMS. Requests can be directed via email to crc@tums.ac.ir. Please note that all new studies will require additional approval from the relevant Research Ethics Committee of TUMS in Iran.

Thank you for your consideration.

Best Regards;

Kazem Zendehdel

Corresponding author

Reviewer #1:

The study utilizes a robust methodology to assess survival disparities in non-metastatic colorectal cancer patients across different types of hospitals in Iran. The focus on high-volume teaching versus non-teaching hospitals provides valuable insights into the impact of hospital type on patient outcomes.
Comments:

1. Some parts of the manuscript are a bit confusing and could be better structured. For instance, the introduction should give a clearer and shorter summary of the research problem and goals via disparities.

**Response:** Thank you for your suggestion. We revised the introduction and clarified the research gap and goals.

2. The manuscript mentions the importance of the study, but it would be helpful to have a more in-depth discussion of other studies about colorectal cancer care differences in poorer countries. This would provide a stronger foundation for the research.

**Response:** As we addressed in the introduction, to our knowledge, no study has evaluated the quality of care in poorer countries so far.

3. It's great that you included different types of hospitals, but it would be good to explain in more detail why you chose these specific hospitals and how they represent the overall healthcare system.

**Response:** As an initial step, research studies like this have been voluntarily conducted by various centers to evaluate the quality of their care as part of routine practice, even years before a comprehensive framework for cancer care quality was developed. These initiatives represent an essential first step in assessing and enhancing the quality of cancer care, mainly when the appropriate organizations in the country have not yet established national systems and oversight mechanisms for such evaluations.

We focused on selecting hospitals prepared to collaborate and participate in this research. While our findings' results and internal validity are solid, we acknowledge that they may not be generalizable to the entire country. We have addressed this limitation in our discussion.

4. The information about the variables is thorough, but it would be useful to clarify exactly how you measured each variable and gathered the data. This would make it easier for others to repeat your study.

**Response:** We carefully addressed it in the "Variables" section.

5. The TMLE method is complex, so it would be helpful to explain it more clearly.

**Response:** We added a further description of the TMLE method. See lines: 231-242

6. Please explain why you chose the number of participants you did and if you calculated how many you needed to get reliable results (power analysis).

**Response:** We included all eligible patients admitted to the hospitals during the study period, ensuring that no cases were selectively excluded. While the sample size is adequate, we faced limited power in subgroup analyses, particularly rectal cancer cases in the NTNC hospital. However, the significant differences in patient survival suggest we had a sufficient minimum number of patients for these analyses. We acknowledge that a larger sample size would yield more robust results and recognize this as a limitation in our discussion.

7. The discussion does a good job of putting the results in context, but it could go deeper into why the differences in care exist.

**Response:** We enhanced the discussion in response to this comment, emphasizing how the differences between types of hospitals can lead to disparities in patient outcomes.

8. Based on your findings, please suggest specific actions or changes that could be made to improve the situation (or in further studies).

**Response:** We added more suggestions in the last part of the discussion and the conclusion sections. We believe that health policymakers and authorities should leverage the educational and scientific frameworks established in TCCS and provide support and resources to non-cancer facilities that may enhance the quality of cancer care across various healthcare settings. We also suggested scaling up this study by including additional centers to create comprehensive evidence regarding the quality of cancer care nationally and improve the national cancer control program.

**Reviewer #2:**

In the present study, the authors evaluated the outcomes and prognosis of colorectal, colon, and rectum cancers by the level of care provided in teaching and non-teaching non-cancer hospitals, using a data registry in Iran. The study subject is interesting. However, the use of language should be improved. Additionally, there are further comments and suggestions provided below:

**General comments**
1. There are some typos and grammatical issues within the text. Please proofread the entire manuscript.

**Response:** We proofread the entire manuscript and fixed the errors and typos.

2. Define abbreviations upon their first use in the text.

**Response:** We defined the abbreviations as suggested.

3. Use p<0.01 instead of p=0.00.

**Response:** Thanks. We corrected typos in presenting the P value.

**Abstract**
1. Please clarify and elaborate more on the background and objective of the study.

**Response:** We revised the background as suggested.

2. Check the number: RR=1.39, 95% CI: 1.01-2.022. Should it be 2.02 or 2.022? Revise it in the text if necessary.

**Response:** Thanks for noting this. We corrected the typo.

3. Revise the keywords based on MeSH.

**Response:** We updated the keywords based on MeSH terms.

**Introduction**
1. The SEER program in the US can also be discussed.

**Response:** We added results from the SEER program in the introduction.

2. In the second paragraph, please elaborate more and mention some of the findings from countries that have subnational or hospital-level data.

**Response:** We revised the introduction and incorporated the reviewer's suggestion in the new version.

**Methods**
1. Provide the exclusion criteria under the "Patients" subheading.

**Response:** We added the exclusion criteria as suggested. Please see lines: 148-153

2. Cite the relevant references for the guidelines used in the study (Variables subheading).

**Response:** We cited the relevant references for the guidelines used in the study.

3. It is recommended to provide an "Outcome" subheading and define the outcomes of interest for this study.

**Response:** We defined the outcome variables in the "Outcome" subheading line: 206-209

**Results and tables**

1. Table 1: Define what you mean by high, average, and low insurance coverage, and how you determine the risk of surgery. Also, it might be helpful to provide a column to represent the p-value between the groups for each variable.

**Response:** Thank you for the reminder; we have added the definitions of these two variables in the methods section under the "Variables" subheading. Regarding the p-value column in Table 1, we followed the STROBE guidelines, which recommend against reporting p-values for descriptive tables.

The statement in the STOBE guideline is:

*"Inferential measures such as standard errors and confidence intervals should not be used to describe the variability of characteristics, and significance tests should be avoided in descriptive tables. Also, P values are not an appropriate criterion for selecting which confounders to adjust for in analysis; even small differences in a confounder that has a strong effect on the outcome can be important"*

*Ref: Vandenbroucke JP, von Elm E, Altman DG, Gøtzsche PC, Mulrow CD, Pocock SJ, Poole C, Schlesselman JJ, Egger M; STROBE Initiative. Strengthening the Reporting of Observational Studies in Epidemiology (STROBE): explanation and elaboration. Int J Surg. 2014 Dec;12(12):1500-24. doi: 10.1016/j.ijsu.2014.07.014. PMID: 25046751.*

2. You mentioned that comorbidities were also considered. Please provide the baseline characteristics for comorbidities in Table 1 and clarify which comorbidities were considered.

**Response:** Thank you for your comment. It was a typo in our paper. In fact we used the "risk of surgery". Therefore, we replaced comorbidities with "risk of surgery".

Reviewer #3:

This study addresses a critical gap in understanding the disparities in care quality across different hospital types in the Eastern Mediterranean region. Below are my general comments and specific recommendations:

General comments:
1. The objective of the study, whether it focuses on the quality of care or prognosis, needs to be stated clearly at the outset.

**Response:** Thank you for your review and comments. While some steps and findings of this study mirror those of prognostic factor research, our primary focus is on evaluating care quality and treatment outcomes across various hospital groups.

We clarified this in the paper.

2. The rationale and importance of this study, as well as its contribution to the body of knowledge on colorectal cancer care quality, need to be more clearly articulated.

**Response:** We revised the introduction and addressed the concern raised by reviewer.

3. Ensure that the outcome measures and study setting are explicitly defined.

**Response:** We clarified the outcome definition in the methods section and devopted specific subheading for it.

4. Maintain consistent language and style throughout the manuscript.

**Response:** We proofread the entire manuscript.

5. Improve the writing style by starting with a stem statement followed by key points relevant to the study's objectives and direction.

**Response:** We revised the manuscript to enhance the style and ensure the consistency of concepts."

Specific sections:
Abstract: Please revise the abstract to reflect the recommendations provided for the entire article.
**Response:** We revised the abstract according to the suggestion.

** Introduction**
1. Include a section that describes the burden of colorectal cancer and the significance of monitoring and evaluating care outcomes.

**Response:** We added this information in the first paragraph of the introduction.

2. Justify the selection of input, process, and outcome indicators commonly used to monitor care quality, enabling readers to understand the global and regional context. This will help readers understand the international and regional context of the study.

**Response:** We made the necessary revisions and provided a comprehensive framework for the study, including the essential input, process, and output factors or indicators considered in the model. Figure 1 in the revised paper illustrates this framework clearly.

3. Follow statements such as "registry-based study has been advanced by a collaborative network" with an explanation of the network's function and contribution to improving care quality.

**Response:** We have carefully addressed it lines 110-114.

4. Describe the multidisciplinary team involved, the characteristics of well-equipped hospitals, and how these factors contribute to care quality.

**Response:** We added a description for the multidisciplinary team involved and other necessary information. (see lines 92-99).

5. Provide information on existing patient outcomes, survival rates, and process indicators used in colorectal cancer surgery. Discuss the advantages and disadvantages of these indicators.

**Response:** We addressed them in lines 72-81, respectively.

6. Include evidence that patient outcomes for colorectal cancer vary based on hospital type (teaching status, cancer specialization) and location.

**Response:** We added some evidence in the manuscript (please see lines 333-342).

7. Clearly describe the infrastructure gaps relevant to colorectal cancer care quality that the study aims to highlight.

**Response:** In this study we addressed the input, process and outome in care quality. In input, we considered some important factors in patient and tumor side, but presumed the hospital infrastructures are not significantly different in two types of hospitals as mentioned in lines 171-174.

8. Refine the objective statement using the SMART criteria (Specific, Measurable, Achievable, Relevant, Time-bound).
**Response:** Thanks for such a constructive comment. We revised the study objective accordingly.

** Methods**
1. Provide an overview of the healthcare systems in Iran, particularly colorectal cancer services, and any changes in service delivery over time.

**Response:** We added a parsgraph about colorectal cancer care in Iran (91-100).

2. Organize the Methods section to provide a clear contextual understanding of colorectal cancer treatment in Iran, followed by the role of different hospitals, the quality-network, and data collection processes. Conclude with detailed data management and analysis descriptions.

**Response:** We revised the methods section as recommended.

3. Clarify whether QRN-CRC is limited to specific hospitals or is a nationwide program. Provide the rationale for selecting the quality registry network and differentiate between hospital types.

**Response:** As an initial step, research studies like this have been voluntarily conducted by various centers to evaluate the quality of their care as part of routine practice, even years before a comprehensive framework for cancer care quality was developed. These initiatives represent an essential first step in assessing and enhancing the quality of cancer care, mainly when the appropriate organizations in the country have not yet established national systems and oversight mechanisms for such evaluations.

We focused on selecting hospitals prepared to collaborate and participate in this research. While our findings' results and internal validity are solid, we acknowledge that they may not be generalizable to the entire country. We have addressed this limitation in our discussion.

4. Explain how clinical registry data is collated, whether through hospital information systems or retrospective patient tracking and case note reviews.

**Response:** We provided this information and clarified the data collection procedure in a specific subheading named "Database" in the revised paper. Please check lines 155-167.

5. Specify the number of hospitals included in the study, the definition of high-volume hospitals, and the quality of data in the cancer registry.

**Response:** We provided details about the hospitals in the revised manuscript.

6. Summarize variables in a table with clear definitions to aid reader comprehension. Include guidelines for patient treatment based on cancer stages as appendices, if necessary.

**Response:** We completed the "variables" subheading and provided a figure for the framework of the variables used in this study.

7. Clearly define the study's outcome or dependent variables.

**Response:** we clarified the definition of the outcomes as the dependent variables at the end of the "variables" subheading.

8. Clarify the study duration and follow-up period. Provide details on patient follow-up timelines to help readers understand the duration of follow-up.

**Response:** We added the median of follow-up in this study. In addition, we provided a supplementary table and compared the censorship by different variables. (see Appendix Table 1)

9. Describe potential selection bias reasons and the steps taken to mitigate it.
**Response:** We compared the censored and non-censored patients ((see Appendix Table 1) and showed that there were no significant differences in each group according to different variables,

**Results**
1. Summarize the patient flow in a diagram to better illustrate study inclusion and exclusion processes

**Response:** Thank you for recognizing the importance of the "patient flow diagram" for this study and for your feedback. We added this diagram in a supplementary file.

2. If mortality rates are low, compare them with international studies and discuss potential factors contributing to these rates in Iran – in discussion

**Response:** Thank you for your comment, but we were not sure that this comparison can be addressed in this manuscript with different scopes and objectives of this paper. For an international comparison, we will need to measure net survival using the relative survival method. We provided such a comparison from 15 cancer subsites in another paper from our team:

***Nemati S, Saeedi E, Lotfi F, Nahvijou A, Mohebbi E, Ravankhah Z, Rezaeianzadeh A, Yaghoobi-Ashrafi M, Pirnejad H, Golpazir A, Dolatkhah R, Alvand S, Ahmadi-Tabatabaei SV, Cheraghi M, Weiderpass E, Bray F, Coleman MP, Etemadi A, Khosravi A, Najafi F, Mohagheghi MA, Roshandel G, Malekzadeh R, Zendehdel K. National surveillance of cancer survival in Iran (IRANCANSURV): Analysis of data of 15 cancer sites from nine population-based cancer registries. Int J Cancer. 2022 Dec 15;151(12):2128-2135. doi: 10.1002/ijc.34224. Epub 2022 Aug 6. PMID: 35869869.***

3. Explain how deaths were attributed to colorectal cancer or competing risks, and describe the data collection method for death information.

**Response:** As we explained in the methods of following up in this study, evaluation of the competing risk of death will be unavailable in some of these methods and some groups of patients. Other causes of mortality have been checked in the phone call interview, and we found 13 patients in this group with other mortality causes, and there was no significant difference between the two groups of TCCs and NTNCs. Therefore, we think that competing risks are not an issue in this paper.

4. Discuss the appropriateness of different outcomes like 1-year survival or 5-year survival to monitor colorectal cancer.

**Response:** We added the early mortality rates (30, 36, 90, 180 days mortality) beyond the five-year survival as outcome quality indicators and compared TCCs and NTNCs. We also explained the logic and importance of these indicators in the discussion section.

5. Specify if the study focuses on patient-related factors or healthcare service factors.

**Response:** We discussed this issue in the framework of the study added as Figure 1.

**Discussion**
1. Ensure alignment between methods, results, and discussion sections. The term "prognosis" should be introduced consistently throughout the manuscript.

**Response:** We consistently incorporated the study keywords, specifically 'quality of care and patient outcome,' throughout all sections of the manuscript.

2. Focus on variables included in the study and discuss their relevance.

**Response:** We revised the Discussion, accordingly.

3. Discuss whether the study findings are still applicable in the current context, considering healthcare advancements since the study period.

**Response:** We did not experience major change in the mangent of the colorectal cancer patients since the study period. We believe that the findings are still relvant in the current context.

4. Align the discussion on socioeconomic status with the study's focus on care quality.

**Response:** We updated the discussion and addressed the reivwers concern. The lines between 1048 and 1058 discusse the SES and care quliaty. Please see the lines: 355-364.

5. Clarify the collaborative program's strengths and its implementation.

**Response:** we discussed the strength and limitations in lines 375-379 and 386-394.

6. Be consistent about sample size adequacy and its implications for the study's findings.

**Response: We** included all eligible patients and avoided the selection or exclusion of certain cases. although the sample size, particularly for rectal cancer in NTNC hospitals, is small, a significant difference in patient survival was observed.

7. Clearly describe the study setting early in the manuscript for better contextual understanding.

**Response:** We added a figure to clarify the study framework. Please see Figure 1.

8. Cross-reference and label figures appropriately.

Response: We carefully checked the cross reference and labeled figures.

**Minor points:

Provide full spellings followed by abbreviations in parentheses.

**Response:** We fixed this for all abbreviations.

Ensure consistent language and tone throughout the manuscript to avoid the appearance of sections written by different authors.

**Response**: We revised the manuscript to ensure consistency of language and tone.

Reviewer #4:

In this study, the authors assess the differences in the survival of patients with colorectal cancer in two types of hospitals in Iran and draw conclusions about the quality of care. While the subject is interesting, there are some important conceptual issues that the authors must explain in their methodology before reaching any conclusions.

The title is confusing because it claims to investigate the quality of care in developing countries, whereas the study is conducted only on hospitals in Iran. It is incorrect to generalize the performance of Iranian hospitals to all developing countries. The abstract and title are misleading. The term "quality of care" is broad and can be used for various purposes. It would be better if the authors clarified that they are comparing the survival of colon and rectal cancer patients in two types of hospitals. It is questionable whether the differences between these two types of hospitals can serve as an indicator of quality of care, given that many hospital variables are ignored in this study.

The most important issue is that the authors must clearly state the names of the hospitals and the cities where they are located in their methodology, because there are a lot of variables which could affect the quality of care related to hospitals. One significant flaw in this study is that it includes only one NTNC and three TCC hospitals, yet attempts to generalize the performance of just four hospitals to all of Iran and developing countries. Furthermore, there is no clear definition of these hospitals in the text (e.g., high-volume?). Important variables about the hospitals, such as the number of physicians, nurses, and beds, are missing. Due to these flaws, I believe the article is not suitable for publication in its current format. The authors need to revise the title, abstract, and content of the study to more accurately reflect what they actually did. Additionally, they must provide detailed information about the hospitals included in the study.

**Response:**

Thank you for your review and comments. We have revised the manuscript's title, abstract, and content to address the issues raised by the reviewers. We acknowledge that our study included only one non-teaching, non-cancer center (NTNC) and three teaching cancer centers (TCCs). Despite this limitation, as discussed in the paper, we believe it still provides valuable evidence regarding disparities in the quality of care between NTNCs and TCCs in a country like Iran. We have included additional details about the hospitals involved, but we had to consider ethical implications when providing specific information and the names of the hospitals.

Regarding the definition of high-volume hospitals for colorectal cancer (CRC) surgery, as clarified in the text, we established that a high-volume center is one that treats approximately 100 non-metastatic CRC patients per year, noting that there is no universally accepted threshold for this classification.

**"**Some minor issues:

- Note that the p-value cannot be equal to 0. Please change all instances of p-value = 0.00 to something like p-value < 0.001.

**Response:** We corrected the typo. Thanks.

- Please mention in your methodology if there are any missing values and how you handled them.

**Response:** We clarified this issue in the methods section. Although the amount of missing data was small, it will not affect our results. We described that the patients with missing values were included as a separate group in the variables included in the statistical models.

- On pages 8 and 14, where you explain and use TMLE, please clarify which hospital type is considered as the reference in calculating the RR.

**Response:** We clarified that the TCC group was the reference group in the TMLE model.
