# Supplementary material for: “Colorectal Cancer Care Quality in a Developing Country: Insights from a Comparison of Teaching and Non-teaching Hospitals in Iran”
Source: PLoS One. 2025 Sep 8;20(9):e0326796. doi: 10.1371/journal.pone.0326796 (PMC12416677; doi:10.1371/journal.pone.0326796)
Supplement: Attachment — Submitted filename: Rebuttel Letter_D-23-43777_2nd_revision.docx [file pone.0326796.s004.docx]

Kazem Zendehdel,

Professor at the Cancer Research Center,

Cancer Institute, Tehran University of Medical Sciences,

Tehran, Iran.

[kzendeh@tums.ac.ir](mailto:kzendeh@tums.ac.ir)

02-26-2025

Dear Dr. Sina Azadnajafabad,

We would like to express our gratitude to you and the reviewers for the insightful comments and suggestions on our manuscript titled "Bridging Gaps in Colorectal Cancer Care Quality in Developing Countries: Navigating Disparities with the Right Approach, Analytical Methods, and Minimum Essential Data (PONE-D-23-43777)."

We have carefully addressed each reviewer's comments and made the necessary revisions throughout the manuscript. In response to the constructive feedback from the four reviewers, we undertook extensive revisions in “introduction” and “discussion” sections. We are very pleased that, with the reviewers' feedback, we were able to revise our study manuscript in a more precise and clear manner. We provide highlighted and clean versions of the revised paper for your consideration. We hope the revised manuscript will be accepted for publication in PLOS ONE.

Thank you for your consideration.

Best Regards;

Kazem Zendehdel

Corresponding author

1- Reviewer #1: The revision is comprehensive. However, I suggest the authors to elaborate deeper into the implications of selection bias and unmeasured confounders as the potential limitation of study.

**Response:** See lines: 401-408

2- Reviewer #3: The authors have attempted to address my concerns and feedback; however, critical issues remain regarding the focus and objectives of the study and the added value it contributes to improving quality of care for colorectal cancer (CRC). While the authors conclude that care in teaching hospitals is better than in non-teaching hospitals based on hazard ratios, this finding alone does not sufficiently establish the broader importance or contribution of the study.

**Response:** We have thoroughly revised the introduction and objectives of the manuscript and have added the necessary explanations.

3- The rationale and importance of the study remain insufficiently addressed. The authors have not clearly articulated how their findings contribute to the body of evidence on CRC care quality. Specifically, the study does not explore what actionable insights can be drawn to improve care in non-teaching hospitals, nor does it contextualise findings within existing literature

**Response**: Regarding the rationale and importance of the study we added more useful explanations in the introduction part of the manuscript. Additionally, the necessary explanations regarding which aspects of the healthcare system non-academic hospitals should enhance in the management of colon and rectal cancers to reduce the existing differences in patient survival have been added in the Discussion section, lines 338–345.

4- The focus on teaching status as the sole determinant of care quality oversimplifies a complex issue. It neglects to consider other critical factors such as healthcare system organisation, resource allocation, or regional disparities.

**Response**: In addition to what has been stated in the introduction of the manuscript, health system orientation, resource allocation, and regional disparities are important in evaluating cancer care quality. According to the methodology of healthcare quality assessment, factors such as structural aspects and resource allocation are considered as inputs. When process evaluation in healthcare is conducted using quality indicators and outcome assessment, identifying disparities and differences can justify further examination of inputs to determine the underlying causes of these variations (see lines 412-415). Naturally, incorporating all these factors into a single model within this study is not feasible, as it would complicate the understanding of causal relationships.

5- The authors cite the STROBE guidelines, which recommend avoiding p-values in tables describing variability of characteristics. However, the text includes a lengthy description of differences based on p-values. This inconsistency undermines the methodological clarity and should be addressed. The authors should clearly define the purpose of presenting p-values in the study and align their reporting with the cited guidelines.

**Response**: We revised tables and text and described differences based on confidence interval or p-value.

6- The first objective—assessing quality of care independent of hospital type—is only partially addressed. The manuscript predominantly focuses on outcome measures (e.g., mortality and survival rates) without providing a comprehensive assessment of quality of care. Key dimensions such **as safety, person-centered care, and accessibility are absent**.

**Response**: The “introduction” and “discussion” sections of the manuscript have been revised and we addressed our approach to assessment of cancer care quality and rationale of this study. According to the definition provided in the manuscript, the primary component of quality evaluated in this study is “effectiveness,” while other components—such as “acceptability,” “equity,” “optimality,” and “legitimacy”—are undoubtedly important but fall outside the scope of our study.

7- The assumption that hospital infrastructure is comparable is problematic. The authors acknowledge differences in infrastructure (e.g., advanced diagnostics, multidisciplinary teams in teaching hospitals) but do not explore how these differences affect quality. While adherence to guidelines is included, this alone does not represent a holistic evaluation of care quality.

**Response**: We have updated the Introduction section to thoroughly explain the study's rationale and approach. Initially, our quality assessment focuses on process indicators—such as adherence to clinical guidelines—and their impact on patient outcomes. Once we identify any outcome disparities, after controlling for potential confounders, we plan to delve deeper into the surgical department by evaluating its specific quality indicators. Furthermore, comparing hospital infrastructures may help reveal the underlying causes of these differences. Believing that structural factors influence process measures, which in turn affect outcomes, we designed our causal model to distinctly separate structural and process variables.

8- The second objective—comparing high-volume hospitals and comprehensive cancer centres—requires further clarification and depth. I suggest that the authors explicitly address the following assumptions:
   Quality of care is determined by hospital type. Please provide evidence from existing literature supporting the premise that teaching hospitals deliver better care due to multidisciplinary teams, structured adherence to guidelines, and access to advanced resources; and discuss why non-teaching hospitals, despite being high-volume, may lack comparable capabilities.
   Hospital infrastructure and resources are comparable within categories. Clarify whether the included hospitals are truly representative of their respective categories. The authors assume that disparities are due to teaching status, yet they do not evaluate or account for unmeasured factors such as staffing, technology, or hospital policies.

**Response**: Thank you for your suggestion. We have revised the "Introduction" section of the manuscript, incorporating a rational and historical approach in the literature review to ensure that all aspects of the suggested notes—regarding hospital categorization, infrastructure comparisons, and representativeness—are clearly addressed.

9- Differences in quality can be measured by outcomes. Survival rates, guideline adherence, and process indicators (e.g., emergency surgeries) are valid proxies for quality of care, but the authors should acknowledge their limitations in fully capturing care quality dimensions.

**Response:** We revised the introduction totally and also add a paragraph to the study limitation part of the “discussion” section (see lines 412-418).

10- Patient profiles are comparable after adjustment. Although adjustments were made for confounders, the possibility of bias remains, particularly due to differences in patient selection (e.g., more emergency cases in non-teaching hospitals). This limitation should be explicitly discussed.

**Response:** Thank you for this suggestion. In the limitations section of the Discussion, we explain that only the minimum essential dataset needed for a comprehensive comparison of hospitals was included (Please see lines: 406-408).

11- Discussion and Contextualisation. The manuscript highlights disparities in outcomes between hospital types but does not explore the underlying causes or contextualise these disparities meaningfully. For instance: What specific processes or resources in teaching hospitals lead to better outcomes? How do these findings align with global evidence on CRC care disparities, particularly in similar healthcare systems? I recommend that the authors expand the discussion to address these gaps and provide actionable recommendations for improving care in non-teaching hospitals. Provide more description/ data on hospital-specific characteristics, such as staffing levels, resource availability, and patient volumes, to strengthen the comparison.

**Response:** We have revised the Introduction section to comprehensively detail the study’s rationale and approach. As a first step in assessing quality of care, our focus is on process indicators—such as adherence to clinical guidelines—and their impact on patient outcomes. Once we identify any outcome disparities while controlling for potential confounders, we plan to further investigate the surgical department by examining its specific quality indicators. For instance, approved indicators related to the surgical process include achieving a dissection of at least 12 lymph nodes during colon cancer surgery and ensuring an interval of 8–12 weeks from the end of neoadjuvant chemoradiation therapy to the rectal cancer surgery. Additionally, comparing hospital infrastructures may help uncover the root causes of these differences. We have applied these strategies in separate studies. We added a paraghraph based on this pathway in the “discussion” section.

12- Discuss the generalisability of findings, particularly given the small sample of hospitals included.

**Response:** The proposed quality assessment model was developed based on a minimum essential dataset while considering the most influential and confounding factors affecting the outcomes of colorectal cancer surgery patients in two hospital groups. By evaluating the model’s feasibility and by encouraging the centers to implement the model and assess the outcomes derived from data collection, a disparity between the two hospital groups was ultimately observed, thereby enabling the generaliability of the model under similar conditions. It is important to note that the generalizability of findings similar to those in this study was not the primary objective.
