# Supplementary material for: “Colorectal Cancer Care Quality in a Developing Country: Insights from a Comparison of Teaching and Non-teaching Hospitals in Iran”
Source: PLoS One. 2025 Sep 8;20(9):e0326796. doi: 10.1371/journal.pone.0326796 (PMC12416677; doi:10.1371/journal.pone.0326796)
Supplement: Attachment — Submitted filename: Rebuttel Letter_D-23-43777_3rd_revision.docx [file pone.0326796.s005.docx]

Kazem Zendehdel,

Professor at the Cancer Research Center,

Cancer Institute, Tehran University of Medical Sciences,

Tehran, Iran.

[kzendeh@tums.ac.ir](mailto:kzendeh@tums.ac.ir)

05-05-2025

**Rebuttel Letter**

In response to the feedback received, we have thoroughly addressed the remaining reviewer comments and have made the necessary revisions to the manuscript. We are submitting both clean and highlighted version of the revised manuscript.

Best Regards;

Kazem Zendehdel

Corresponding author

6. Review Comments to the Author:  the revised objectives remain broad and somewhat disjointed from the methods and results. I recommend refining the objectives to clearly focus on comparing survival outcomes and quality indicators between hospital types. Broader aims, such as registry development and quality model proposals, should be framed separately as background or future directions.

**Response:** Thank you for your comment. We have revised the objectives section to focus on the specific findings of the study.
